# Optimal UAV's Deployment and Transmit Power Design for Two Users Uplink NOMA Systems

**DOI:** 10.3389/fnbot.2020.599344

**Published:** 2021-01-15

**Authors:** Fayong Zhao

**Affiliations:** School of Physics and Electronic Engineering, Fuyang Normal University, Fuyang, China

**Keywords:** UAV network, uplink NOMA, power control, UAV's deployment, transmit power design

## Abstract

In order to fully utilize the spectrum resources, this work considers a unmanned aerial vehicle (UAV) uplink communication system based on non-orthogonal multiple access technology (NOMA), in which the UAV receives information from the ground users with a certain flying altitude. As an initial study, we consider a simplified setup with two ground users to draw some insightful results. Explicitly, we first formulate an optimization problem that maximizes the sum throughput subject to each user's transmit power constraint and their corresponding minimum transmission rate requirement. Then, both the optimal transmit power and UAV's deployment location are derived with the aid of employing the Karush-Kuhn-Tucher (KKT) conditions. Simulation results show that the proposed UAV's deployment scheme with the users' power allocation can achieve a higher sum throughput compared with two existing benchmark schemes.

## Introduction

Non-orthogonal multiple access (NOMA) is one of the key technologies for future wireless networks, which meets the heterogeneous demands on low latency, massive connectivity, high throughput, etc. (Dai et al., 2015; Ding et al., 2017). Technically, NOMA combined several modern wireless technologies, including multiple-input multiple-output (MIMO), massive MIMO and millimeter wave communications was studied in Vaezi et al. (2019) and Wang et al. (2020). Besides, the intelligent reflecting surface (IRS) aided NOMA systems were investigated in recent work (de Sena et al., 2020). The apparent benefit of NOMA which blends those compelling techniques is that it has ability in improving scalability, spectral efficiency and energy efficiency. Compared with traditional orthogonal multiple access (OMA) schemes, such as frequency division multiple access (FDMA), time division multiple access (TDMA) and code division multiple access (CDMA), NOMA simultaneously share the time, frequency and code resources. Consequently, the inter-user interference is introduced actively. Notably, correct demodulation is achieved at the receiver through successive interference cancellation (SIC) (Saito et al., 2013; Chen et al., 2017). In Ding et al. (2014) and Timotheou and Krikidis (2015), downlink NOMA networks were studied, where the authors have demonstrated that NOMA can achieve better outage performance than that of OMA schemes, when both the users' rate and power allocation are carefully designed. In Zhang et al. (2016) and Al-Imari et al. (2014), uplink NOMA networks were discussed, where they showed that the uplink NOMA can improve both the spectrum efficiency and fairness index compared with OMA technique.

The researches related to unmanned aerial vehicles (UAVs) has become a hot topic due to their wide application prospects, such as goods delivery, search and rescue, aerial photography, and telecommunications (Zeng et al., 2016). For example, Jiao et al. (2020) presented an intuitive end-to-end interaction system between a human and an UAV in which the UAV can be commanded by natural human poses. Moreover, a brain-inspired decision-making spiking neural network (BDM-SNN) was proposed in Zhao et al. (2018), which can help UAV making decisions in some tasks. In realistic communications, UAVs can be regarded as aerial stations for serving ground users within certain areas. In order to prolong the network lifetime, energy efficient of UAV networks was studied in Amoiralis et al. (2014), Zeng and Zhang (2017), and Zhan et al. (2018). In addition, the authors of Wu et al. (2019) investigated the fundamental throughput, delay, and energy tradeoffs in UAV networks. Furthermore, the authors in Wu and Zhang (2017, 2018) and Wu et al. (2017) pointed out that the fundamental tradeoff between the delay and the throughput in multi-user UAV networks with OMA. For reducing the access latency and improving the communication quality of UAV-based networks, it is reasonable to graft the NOMA technique into UAV networks, which is termed as UAV-enabled NOMA networks (Sharma and Kim, 2017; Cui et al., 2018; Sohail et al., 2018; Liu et al., 2019; Nasir et al., 2019; Zhao et al., 2019; Do et al., 2020). Against this background, a number of works related to the UAV-enabled downlink NOMA networks have been comprehensively studied in Sharma and Kim (2017), Cui et al. (2018), and Sohail et al. (2018). Specifically, a power allocation scheme that maximizes the sum-rate of the UAV networks for reducing the energy consumption was studied in Sohail et al. (2018). In Cui et al. (2018) and Sharma and Kim (2017), the authors proposed a novel algorithm to maximize the minimum average rate by jointly optimizing the UAV's trajectory and its transmit power. Additionally, an UAV-enabled NOMA network with user pairing was studied in Nasir et al. (2019), where one user having the minimum throughput was maximized. Furthermore, to maximize the sum rate of the ground users, the authors in Liu et al. (2019) studied both the UAV's location and its transmit power. Moreover, the UAV-enabled relay NOMA networks were investigated in Do et al. (2020), and it was demonstrated that full-duplex mode can provide better outage performance than half-duplex mode. However, the extension from the downlink NOMA to uplink NOMA is not trivial because the decoding order of SIC in uplink NOMA is completely opposite to that of downlink NOMA. It is worth mentioning that the aforementioned literatures only considered the downlink scenarios, hence these existing contributions are unsuitable for the uplink scenarios, such as the data collection in the upcoming Internet of Things (IoT).

**Figure 1:**
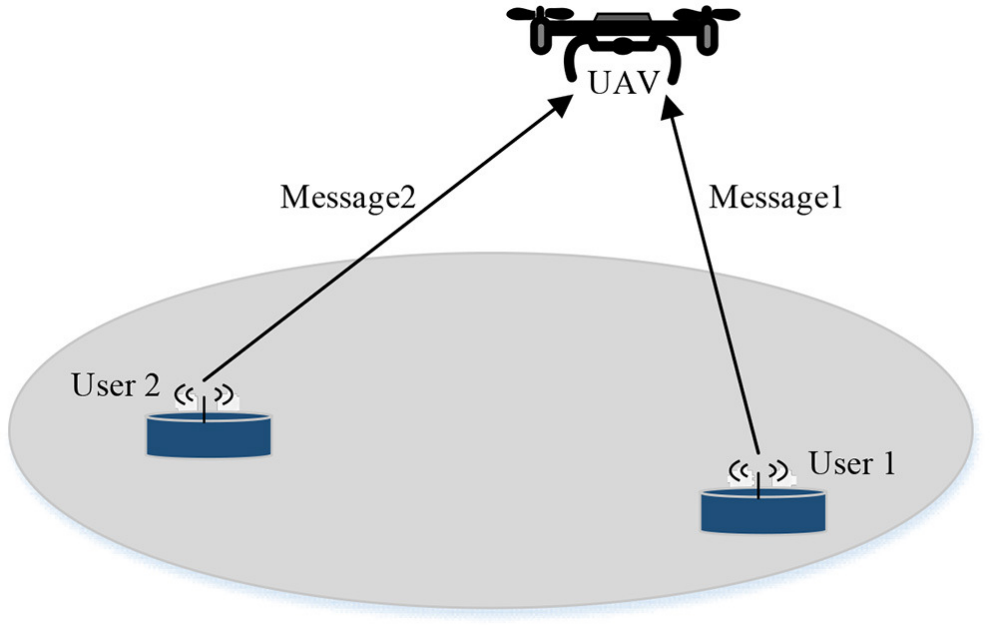
UAV-enabled uplink NOMA network with two users.

Motivated by above-mentioned reasons, this paper considers an UAV-enabled uplink NOMA with power multiplexing network, where an UAV is deployed to collect the messages transmitted from the ground users. We note that UAV-enabled uplink NOMA systems with multi-user is difficult to obtain the optimal design since the formulated optimization problem is generally difficult to tackle directly. As an initial study, similar to Wu et al. (2018), which explores the capacity of UAV-enabled/aided two user communication systems, as shown in Figure 1 we consider the optimal UAV's deployment and each user's power allocation in UAV-enabled uplink NOMA systems with two ground users to get some insightful results. Our goal is to maximize the sum rate by jointly designing the UAV's deployment location and each user's transmit power subject to the transmit power constraints and the quality of service (QoS) constraints. We should point out that our proposed algorithm is significantly different from the recent work (Duan et al., 2019) and (Du et al., 2020). Specifically, Duan et al. (2019) studies the multi-UAV aided uplink NOMA systems, where the transmit power is solved by the proposed SCA-based iterative algorithm, but the UAVs' deployment locations are not optimized. Although Du et al. (2020) designed the UAV deployment location, the proposed algorithm has high computational complexity and only obtains a sub-optimal solution. The main contributions of this work are summarized as follows.

- The analytical solution to the transmit power allocation policy that maximizes the sum rate for the considered dual-user systems is derived. Besides, the result can be further extended to general multi-user systems in a similar way.
- We prove that the optimal UAV deployment location lies on the line segment connected by the two users. Following this fact, the formulated optimization problem is transformed into a univariate quadratic optimization problem. Then the optimal UAV deployment location can be achieved.
- Numerical results confirm the validity of the analytical solution to the optimal UAV deployment location. In addition, our examinations demonstrate that our proposed scheme significantly outperforms the baseline schemes in terms of the sum rate.

The rest of this paper is organized as follows. In section System Model and Problem Formulation we present the system model for an UAV-enabled uplink NOMA network with two users and formulate the optimization problem. The corresponding algorithm for solving problem is introduced in section Proposed Algorithm for Problem (P1). In section Numerical Results, simulation results are provided to demonstrate the performance gain of our proposed algorithm. Finally, our conclusion is provided in section Conclusion.

## System Model and Problem Formulation

In this work, we consider a two user UAV-enabled uplink NOMA network, where the UAV is adopted to collect the messages transmitted from the ground users. We consider a 3-D Cartesian coordinate system where the origin is the geometric center of the two users and the x-axis is the straight line connecting them. Assume that the distances between the two users and the origin as *D*, then the horizontal coordinates of the two users can be denoted as **u**_1_ = [*D*, 0]^*T*^ and **u**_2_ = [–*D*, 0]^*T*^, respectively. It is also assumed that the UAV flies at a fixed altitude *H*, and the horizontal coordinate of the UAV is denoted as ***Q*** = [*x, y*]^*T*^.

To capture the essential characteristics of dual-user UAV-enabled NOMA systems, following Zeng et al. (2019) and the recent 3GPP specification^1^, we assume that the air-to-ground or ground-to-air channel is mainly dominated by line of sight (LoS) link. Thus, the channel gain from user *i*(*i* = 1, 2) to the UAV is given by

(1) $$\begin{array}{l} h_{i}=\sqrt{\frac{\eta_{0}}{\|Q-u_{i}{\|}^{2}+H^{2}}},i=1,2 \end{array}$$

where η_0_ denotes the channel power gain at the reference distance *d*_0_ = 1 m. Since the NOMA transmission scheme is adopted in this work, the received signal at the UAV is a series of superimposed message, which can be expressed as

(2) $$\begin{array}{l} y=\sqrt{P_{1}}h_{1}x_{1}+\sqrt{P_{2}}h_{2}x_{2}+n \end{array}$$

where *x*_1_ and *x*_2_ denote the message transmitted by user 1 and user 2, respectively. *P*_1_ and *P*_2_ are the corresponding transmit power. *n* denotes the zero-mean additive white Gaussian noise (AWGN) with the variance σ^2^ at the UAV. To manage the inter-user interference, the transmit power constraints are given by

(3) $$\begin{array}{l} P_{1}+P_{2}\leq P_{\max}\\ P_{i}\geq 0,i=1,2 \end{array}$$

where *P*_max_ denotes the maximum total transmit power of the two users. For symmetry, we only consider the scenario of *x* ≥ 0 in this work. Consequently, the channel gain of user 1 is greater than that of user 2. According to the principle of NOMA, the SIC is employed at the UAV to decode the messages received from different users. In particular, the UAV first decodes the message from user 1 while treating the message from user 2 as inter-user interference. Then, the decoded message from user 1 is subtracted from the superimposed received signal. Finally, the UAV decodes the message from user 2 without inter-user interference. As a result, the achievable rate of these two users can be expressed as

(4) $$\begin{array}{l} R_{1}=\log_{2}\left(1+\frac{P_{1}{\tilde{h}}_{1}}{1+P_{2}{\tilde{h}}_{2}}\right) \end{array}$$

(5) $$\begin{array}{l} R_{2}=\log_{2}\left(1+P_{2}{\tilde{h}}_{2}\right) \end{array}$$

where ${\tilde{h}}_{i}=\frac{h_{i}^{2}}{\sigma^{2}}=\frac{\zeta_{O}}{\|Q-u_{i}{\|}^{2}+H^{2}}$, and $\zeta_{0}=\frac{\eta_{0}}{\sigma^{2}}$. As a result, the sum rate of the both users is given by

(6) $$\begin{array}{l} R_{sum}=R_{1}+R_{2}=\log_{2}\left(1+P_{1}{\tilde{h}}_{1}+P_{2}{\tilde{h}}_{2}\right) \end{array}$$

Our goal is to maximize *R*_max_ by jointly optimizing the UAV deployment location and the transmit power of the both users with QoS constraints

(7) $$\begin{array}{l} R_{i}\geq r^{*},i=1,2 \end{array}$$

where r^*^ denotes the minimum rate for reliable communication. As a result, the optimization problem can be written as

(8) $$\begin{array}{l} \left(\text{P}1\right):\underset{Q,P_{1},P_{2}}{\max}R_{sum} \end{array}$$

(9) $$\begin{array}{l} s.t.\left(3\right),\left(7\right) \end{array}$$

Problem (P1)^2^ is a non-convex optimization problem due to the non-concavity of the objective function (8) and the non-convexity of the constraint (7), which is, in general, difficult to solve. In the next section, we develop an algorithm to solve this problem.

## Proposed Algorithm for Problem (P1)

This section devises an algorithm to solve problem (P1) based on the solution to the transmit power of both the users. The analytical solution to problem (P1) is given as follows.

### Solution to Transmit Power

Denote Q^*^ as the optimal UAV deployment location and let $h_{1}^{*}$ and $h_{2}^{*}$ be the corresponding channel gains of the user 1 and user 2, respectively. We note that the transmit power optimization problem is a convex problem, which can be efficiently solved the by Lagrangian. More explicitly, the corresponding Karush-Kuhn-Tucher (KKT) conditions are listed as

(10) $$\begin{array}{l} \lambda \geq 0,v_{i}\geq 0,i=1,2 \end{array}$$

(11) $$\begin{array}{l} \lambda \left(P_{1}+P_{2}-P_{\max}\right)=0 \end{array}$$

(12) $$\begin{array}{l} v_{1}\left[r^{*}-\log_{2}\left(1+\frac{P_{1}{\tilde{h}}_{1}^{*}}{1+P_{2}{\tilde{h}}_{2}^{*}}\right)\right]=0 \end{array}$$

(13) $$\begin{array}{l} v_{2}\left[r^{*}-\log_{2}\left(1+P_{2}{\tilde{h}}_{2}^{*}\right)\right]=0 \end{array}$$

(14) $$\begin{array}{l} \lambda -\frac{\left(1+v_{1}\right){\tilde{h}}_{1}^{*}/\ln\;2}{1+P_{1}{\tilde{h}}_{1}^{*}+P_{2}{\tilde{h}}_{2}^{*}}=0 \end{array}$$

(15) $$\begin{array}{l} \lambda -\frac{{\tilde{h}}_{2}^{*}/\ln\;2}{1+P_{1}{\tilde{h}}_{1}^{*}+P_{2}{\tilde{h}}_{2}^{*}}-\frac{v_{2}{\tilde{h}}_{2}^{*}/\ln\;2}{1+P_{2}{\tilde{h}}_{2}^{*}}\\ \;+\frac{v_{1}P_{1}{\tilde{h}}_{1}^{*}{\tilde{h}}_{2}^{*}/\ln\;2}{\left(1+P_{2}{\tilde{h}}_{2}^{*}\right)\left(1+P_{1}{\tilde{h}}_{1}^{*}+P_{2}{\tilde{h}}_{2}^{*}\right)}=0 \end{array}$$

where λ, *v*_1_ and *v*_2_ are the Lagrange multipliers. As per Equation (14), we can obtain

(16) $$\begin{array}{l} \lambda =\frac{\left(1+v_{1}\right){\tilde{h}}_{1}^{*}/\ln\;2}{1+P_{1}{\tilde{h}}_{1}^{*}+P_{2}{\tilde{h}}_{2}^{*}}>0 \end{array}$$

According to Equation (11), we have *P*_1_ + *P*_2_ = *P*_max_. Upon substituting Equation (16) into Equation (15), we have

(17) $$\begin{array}{l} \frac{v_{2}{\tilde{h}}_{2}^{*}}{1+P_{2}{\tilde{h}}_{2}^{*}}=\frac{{\tilde{h}}_{1}^{*}-{\tilde{h}}_{2}^{*}+{\tilde{h}}_{1}^{*}v_{1}}{1+P_{1}{\tilde{h}}_{1}^{*}+P_{2}{\tilde{h}}_{2}^{*}}\\ \;+\frac{v_{1}P_{1}{\tilde{h}}_{1}^{*}{\tilde{h}}_{2}^{*}}{\left(1+P_{2}{\tilde{h}}_{2}^{*}\right)\left(1+P_{1}{\tilde{h}}_{1}^{*}+P_{2}{\tilde{h}}_{2}^{*}\right)}>0 \end{array}$$

where *v*_2_ > 0. As per Equation (13), we have

(18) $$\begin{array}{l} \log_{2}\left(1+P_{2}{\tilde{h}}_{2}^{*}\right)=r^{*} \end{array}$$

Based on the above derivation, the optimal solution to transmit power can be expressed as

(19) $$\begin{array}{l} P_{2}^{*}=\frac{2^{r^{*}}-1}{{\tilde{h}}_{2}^{*}} \end{array}$$

(20) $$\begin{array}{l} P_{1}^{*}\;=P_{max}-P_{2}^{*}. \end{array}$$

### Solution to UAV Deployment Location

To determine the optimal UAV deployment location, we have the following Lemma 1.

Lemma 1. The optimal horizontal coordinate of the UAV for maximizing *R*_*sum*_ should be on the line segment that linked by the two users.

Proof of Lemma 1. Assume Q^*^ = [x^*^, y^*^]^*T*^, (y^*^ ≠ 0), i.e. the optimal deployment location deviates from the line segment. Let us define the achievable rates of the two users at the optimal solution as ${R_{1}}^{*}$ and ${R_{2}}^{*}$, respectively. Then, aided with the results in the above subsection, we can obtain

(21) $$\begin{array}{l} {R_{1}}^{*}=\log_{2}\left(1+\frac{P_{1}^{\text{*}}{\tilde{h}}_{1}^{*}}{2^{r^{*}}}\right)\geq r^{*} \end{array}$$

(22) $$\begin{array}{l} {R_{2}}^{*}=\log_{2}\left(1+P_{2}^{\text{*}}{\tilde{h}}_{2}^{*}\right)=r^{*} \end{array}$$

However, if we deploy the UAV at *Q*′ = [x^*^, 0]^*T*^, the corresponding channel gains ${\tilde{h}}_{1}{\;}^{\prime }\text{ and }{\tilde{h}}_{2}{\;}^{\prime }$ will be larger than ${\tilde{h}}_{1}^{*}\text{ and }{\tilde{h}}_{2}^{*}$ respectively. Assuming that $P_{1}{\;}^{\prime }\text{ and }P_{2}{\;}^{\prime }$ are the optimal transmit power at this time, then we have

(23) $$R_{1}{\;}^{\prime }=\log_{2}(1+\frac{P_{1}{\;}^{\prime }{\tilde{h}}_{1}{\;}^{\prime }}{2^{r^{*}}})\overset{\left(a\right)}{>}\log_{2}(1+\frac{P_{1}^{*}{\tilde{h}}_{1}^{*}}{2^{r^{*}}})=R_{1}^{*}\geq r^{*}$$

(24) $$R_{2}{\;}^{\prime }=\log_{2}(1+P_{2}{\;}^{\prime }{\tilde{h}}_{2}{\;}^{\prime })=r^{*}=R_{2}^{*}$$

where (a) holds since $P_{2}{\;}^{\prime }=\frac{2^{r^{*}}-1}{{\tilde{h}}_{2}{\;}^{\prime }}<P_{2}{\;}^{*}$, and thus having $P_{1}{\;}^{\prime }=P_{\max}-P_{2}{\;}^{\prime }>P_{\max}-P_{2}^{*}=P_{1}^{*}$. As a result, *Q*′ = [x^*^, 0]^*T*^ is also a feasible deployment location. Apparently, the corresponding sum rate will be larger than that at Q^*^.

Similarly, if x^*^>*D*, we can deploy the UAV at *Q*″ = [*D*, 0]^*T*^, and the corresponding sum rate is larger. Consequently, the optimal UAV deployment location has to be located at one point on the line segment linked by the two users. This completes the proof.

Based on the above results, problem (P1) can be further reformulated as

(25) $$\begin{array}{l} \left(\text{P}2\right):\underset{x^{*}}{\max}\phi \left(x^{*}\right) \end{array}$$

(26) $$\begin{array}{l} s.t.0\leq x^{*}\leq D \end{array}$$

where

(27) $$\begin{array}{l} \phi \left(x^{*}\right)=P_{1}^{*}{{\tilde{h}}_{1}}^{*}=\frac{\zeta_{0}P_{\max}}{H^{2}+{\left(D-x^{*}\right)}^{2}}\\ -\frac{\left(2^{r^{*}}-1\right)\left[H^{2}+{\left(D-x^{*}\right)}^{2}\right]}{H^{2}+{\left(D\text{+}x^{*}\right)}^{2}} \end{array}$$

Clearly, problem (P2) is a univariate quadratic optimization problem. The derivative of ϕ(x^*^) is given by

(28) $$\begin{array}{l} \frac{d\phi \left(x^{*}\right)}{dx^{*}}=\frac{2\left[2D-{\left(x^{*}\right)}^{2}-\Gamma x^{*}+\Gamma D-2D\left(H^{2}+D^{2}\right)\right]}{{\left[H^{2}+{\left(D-x^{*}\right)}^{2}\right]}^{2}} \end{array}$$

where $\Gamma =\frac{\zeta_{0}P_{\max}}{2^{r^{*}}-1}$. We note that $\frac{d\phi \left(x^{*}\right)}{dx^{*}}$ is equivalent to

(29) $$\begin{array}{l} 2D{\left(x^{*}\right)}^{2}-\Gamma x^{*}+\Gamma D-2D\left(H^{2}+D^{2}\right)=0 \end{array}$$

It can be observed that Equation (29) is a quadric equation. To proceed, we define Δ = Γ^2^ − 8Γ*D*^2^ + 16*D*^2^(*H*^2^ + *D*^2^). If Δ < 0, ϕ(x^*^) is a monotonically increasing function with respect to x^*^, and therefore the optimal deployment location is arrived when *x*_*opt*_ = *D*. By contrast, as Δ ≥ 0, the two stationary points $x_{1}^{*}$ and $x_{2}^{*}$ can be derived as

(30) $$\begin{array}{l} x_{1}^{*}=\frac{\Gamma -\sqrt{\Delta }}{4D} \end{array}$$

(31) $$\begin{array}{l} x_{2}^{*}=\frac{\Gamma +\sqrt{\Delta }}{4D} \end{array}$$

Based on the relation between $x_{1}^{*}$,$x_{2}^{*}$ and the interval (0, *D*), the solution to the optimal UAV deployment location *x*_opt_ can be obtained via Algorithm 1. Finally, we have to check these obtained solutions until finding the one meets the condition of $R_{1}^{*}\geq r^{*}$.

**Algorithm 1:** Solution to the optimal UAV deployment location.

**Input:** *D*, *H*, r^*^, *P*_max_, and ζ_0_.
1: Calculate $x_{1}^{*}$ and $x_{2}^{*}$ according to Equations (30) and (31), respectively.
2: If $x_{1}^{*}\in \left(0,D\right),x_{2}^{*}\in \left(0,D\right)$, then $x_{\text{opt}}=\arg\max\left\{\phi \left(0\right),\phi \left({x_{1}}^{*}\right),\phi \left({x_{2}}^{*}\right),\phi \left(D\right)\right\}$.
3: If $x_{1}^{*}\in \left(0,D\right),x_{2}^{*}\notin \left(0,D\right)$, then $x_{\text{opt}}=\arg\max\left\{\phi \left(0\right),\phi \left({x_{1}}^{*}\right),\phi \left(D\right)\right\}$.
4: If $x_{1}^{*}\notin \left(0,D\right),x_{2}^{*}\in \left(0,D\right)$, then *x*_opt_ = arg max{ϕ(0), ϕ(*D*)}.
5: If $x_{1}^{*}\notin \left(0,D\right),x_{2}^{*}\notin \left(0,D\right)$, then *x*_opt_ = arg max{ϕ(0), ϕ(*D*)}.
**Output:** The optimal UAV deployment location *x*_opt_.

### Solution to Problem (P1)

**Figure 2:**
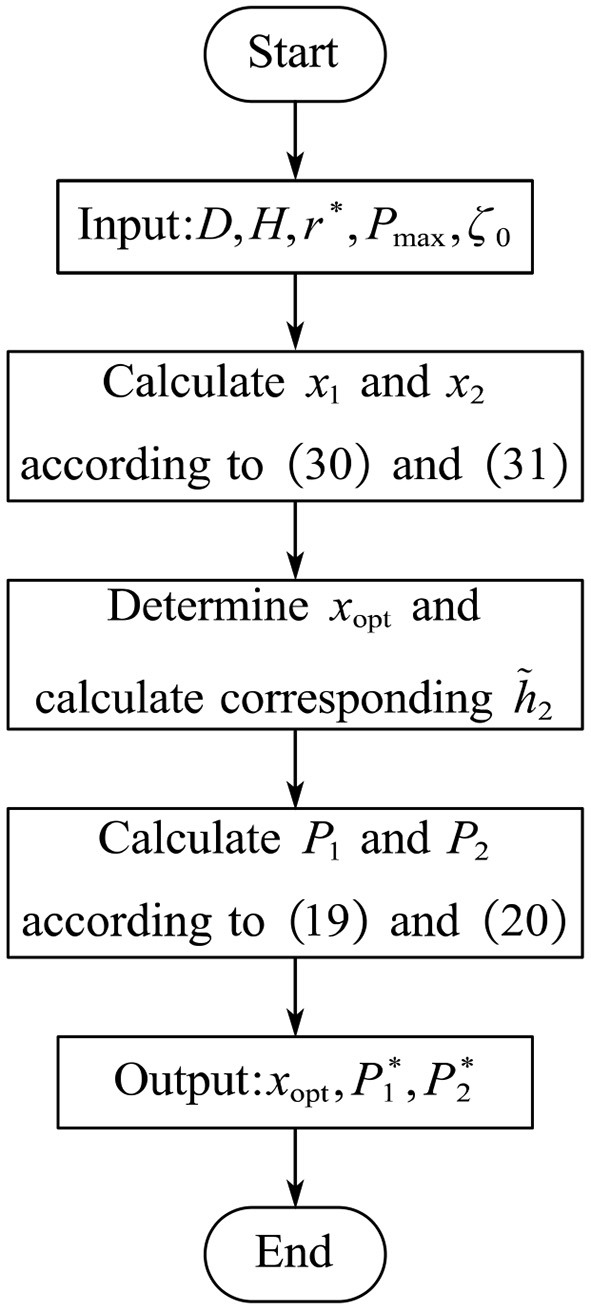
Flow chart of the proposed algorithm.

According to the results of the previous two subsections, the optimal solution to problem (P1) can be obtained via Algorithm 2 given below, and the flow chart of the proposed algorithm is shown in Figure 2. Specifically, the optimal UAV deployment location is first obtained via Algorithm 1. Then, the corresponding channel gains of user 2 is calculated. Finally, the optimal solution to the transmit power can be obtained based on Equations (19) and (20). It is easy to calculate that, in the worst case, the proposed algorithm requires 59 multiplications and 44 additions.

**Algorithm 2:** Solution to Problem (P1).

**Input:** *D*, *H*, r^*^, *P*_*max*_, and ζ_0_.
1: Calculate the optimal UAV deployment position *x*_opt_ via Algorithm 1.
2: Calculate the corresponding channel gain of user 2 ${\tilde{h}}_{2}$.
3: Calculate the optimal transmit power $P_{1}^{\text{*}}$ and $P_{2}^{\text{*}}$ based on Equations (19) and (20).
**Output:** *x*_opt_, $P_{1}^{\text{*}}$, and $P_{2}^{\text{*}}$.

## Numerical Results

In this section, simulation results are provided to demonstrate the effectiveness of our proposed algorithm (denoted as N-LPJO). Referring to the existing the literatures (Wu and Zhang, 2017, 2018; Wu et al., 2017, 2019; Zeng and Zhang, 2017; Cui et al., 2018; Sohail et al., 2018; Zhan et al., 2018), the simulation parameters, unless otherwise specified, are set as: the maximum total transmit power of the two users *P*_max_ = 10dBm, the UAV altitude *H* = 200 m, the distance between the two users and the origin *D* = 400 m, and the reference signal-to-noise ratio (SNR) ζ_0_ = 80 dB.

For comparison, the following three baseline schemes are invoked:

1. FDMA: The UAV collects the messages in FDMA manner, where both the UAV deployment location and transmit power are jointly optimized;
2. N-FLPO: The scheme in Duan et al. (2019), where only the transmit power is optimized while the UAV is fixed at the geometric center of two users, i.e., [0, 0]^*T*^;
3. N-LOFP: The UAV collects the messages in NOMA manner, where only the UAV deployment location is optimized while the transmit power is fixed as *P*_1_ = 2 mW and *P*_2_ = 8 mW.

**Figure 3:**
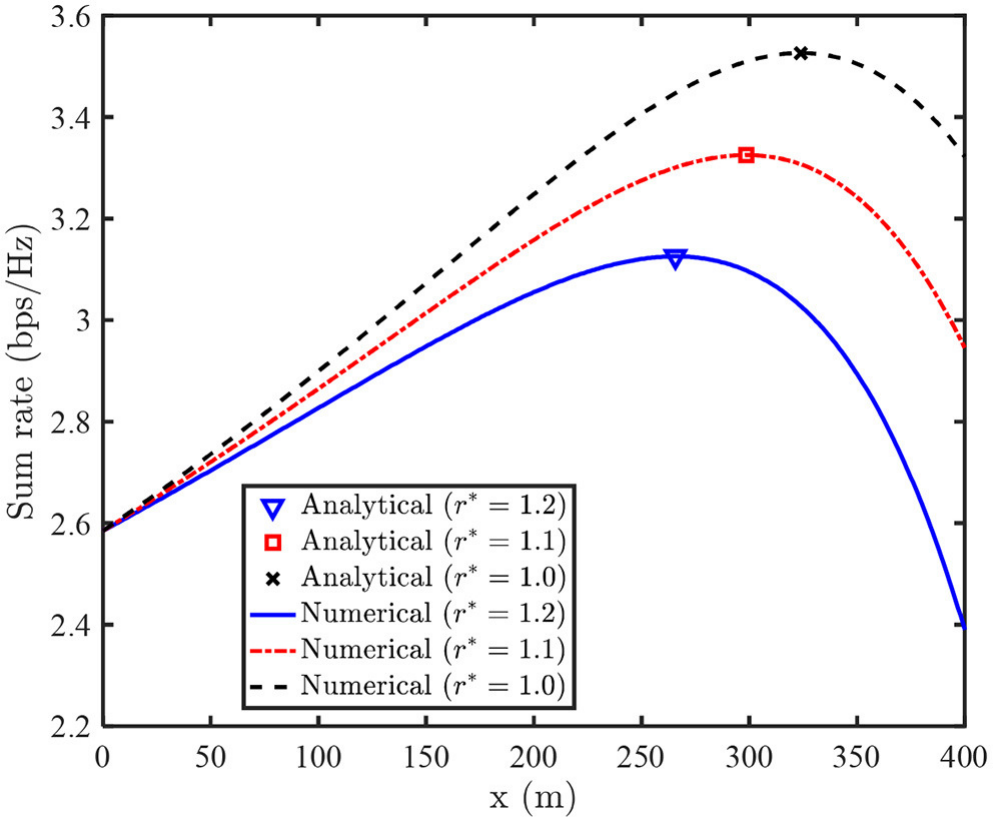
Optimal UAV's deployment location for N-LPJO scheme vs. r^*^.

Figure 3 plots the optimal UAV deployment location of the N-LPJO scheme vs. r^*^, where the numerical results (obtained by 1-D search method) are invoked to reveal the optimality of our proposed algorithm. It can be noted that the sum rates achieved by 1-D search method meets that arrived by our developed analytical solution. This phenomenon implies the optimality of the analytical solution. We also observe that the optimal UAV deployment location is close to user 1, which is beneficial for improving the achievable rate of user 1. Moreover, we observe that the optimal UAV deployment location moves toward the origin as r^*^ increases. This is due to the fact that it will cost less transmit power to meet user 2's QoS constraint if the UAV is deployed close to the origin.

**Figure 4:**
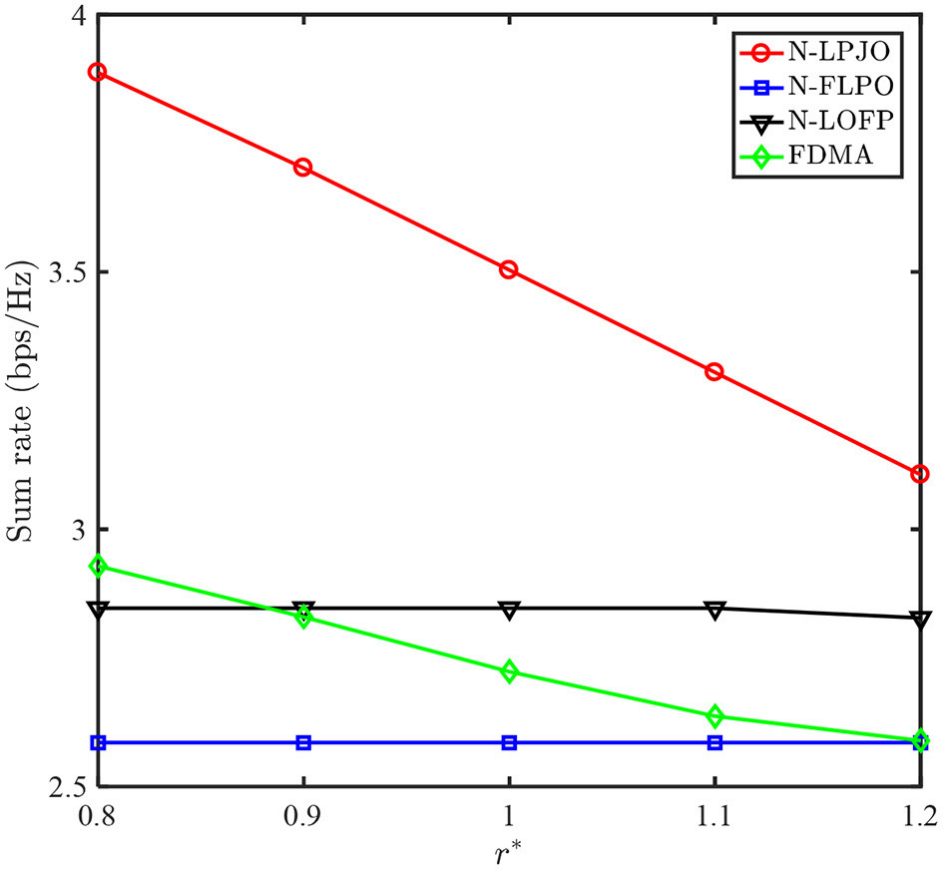
Sum rates of four schemes vs. r^*^.

Figure 4 plots the achievable sum rates of the four different schemes vs. r^*^. We observe that the sum rates of the N-LPJO and the FDMA schemes decreases as r^*^increases. This is mainly because the poorer user has to increase the transmit power for meeting QoS requirement. Meanwhile, the stronger user has to decrease its transmit power. However, the sum rate of the N-FLPO scheme remains unchanged regardless of r^*^, this is due to the fact that ${\tilde{h}}_{1}$ = ${\tilde{h}}_{2}$ and thus $R_{sum}=\log_{2}\left(1+P_{1}{\tilde{h}}_{1}+P_{2}{\tilde{h}}_{2}\right)\;\text{=}\;\log_{2}\left(1\text{+}\left(P_{1}+P_{2}\right){\tilde{h}}_{2}\right)=\log_{2}\left(1+P_{\max}{\tilde{h}}_{2}\right)$ is a constant. Besides, different from the other three schemes, the sum rate of the N-LOFP scheme remains unchanged when r^*^ ≤ 1.1 bps and decreases when r^*^ = 1.2 bps. This is because the optimal UAV deployment location can naturally meet the QoS constraints if the QoS constraints are not very tight. It is clearly shown that our proposed N-LPJO scheme outperforms both the N-FLPO scheme and the N-LOFP scheme, which demonstrates the necessity of optimizing the UAV deployment position and the transmit power, respectively. Furthermore, our proposed N-LPJO scheme also outperforms the FDMA scheme since NOMA can provide higher spectral efficiency than OMA.

**Figure 5:**
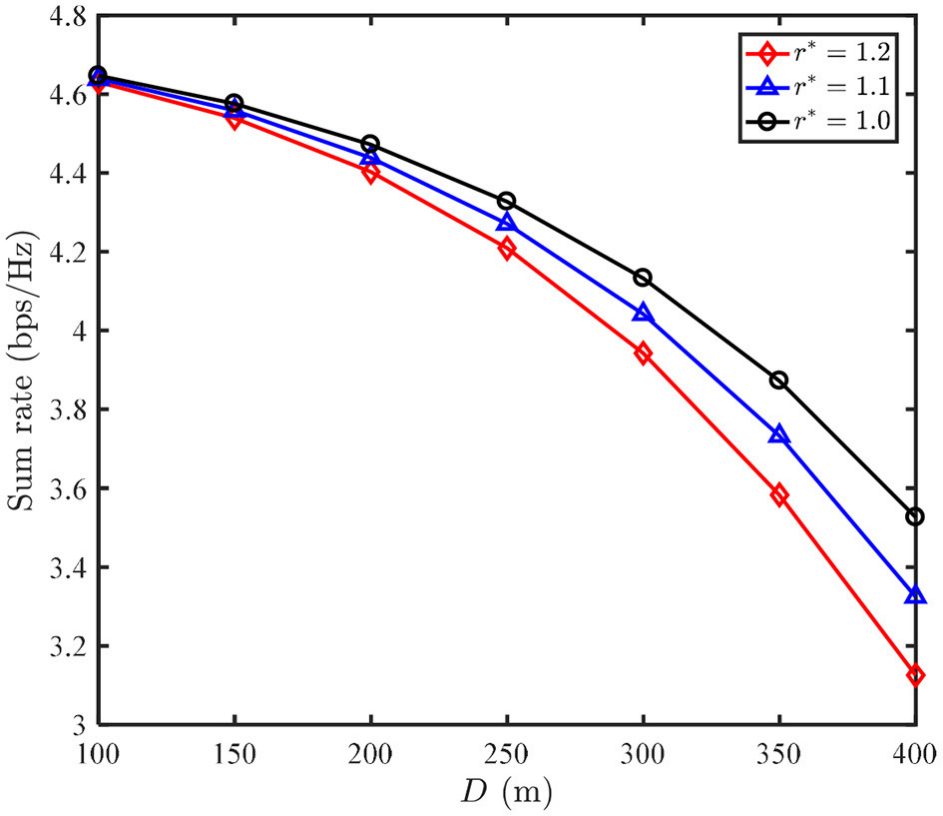
Sum rate of N-LPJO scheme vs. distance D between both users.

Figure 5 shows that the sum rate of our proposed N-LPJO scheme vs. the distance *D* between the two users and the origin. Firstly, we observe that the sum rate decreases as *D* increases for each QoS requirement r^*^. This can be explained as: the channel gain of user 2 sharply decreases once *D* increases. As a result, user 2 has to improve the transmit power to meet the minimum rate requirement, while user 1 has to decrease its transmit power, thus leading to the decrease in the sum rate. Secondly, we note that the difference of the sum rate achieved by the different r^*^ becomes apparent with the increasing of *D*.

**Figure 6:**
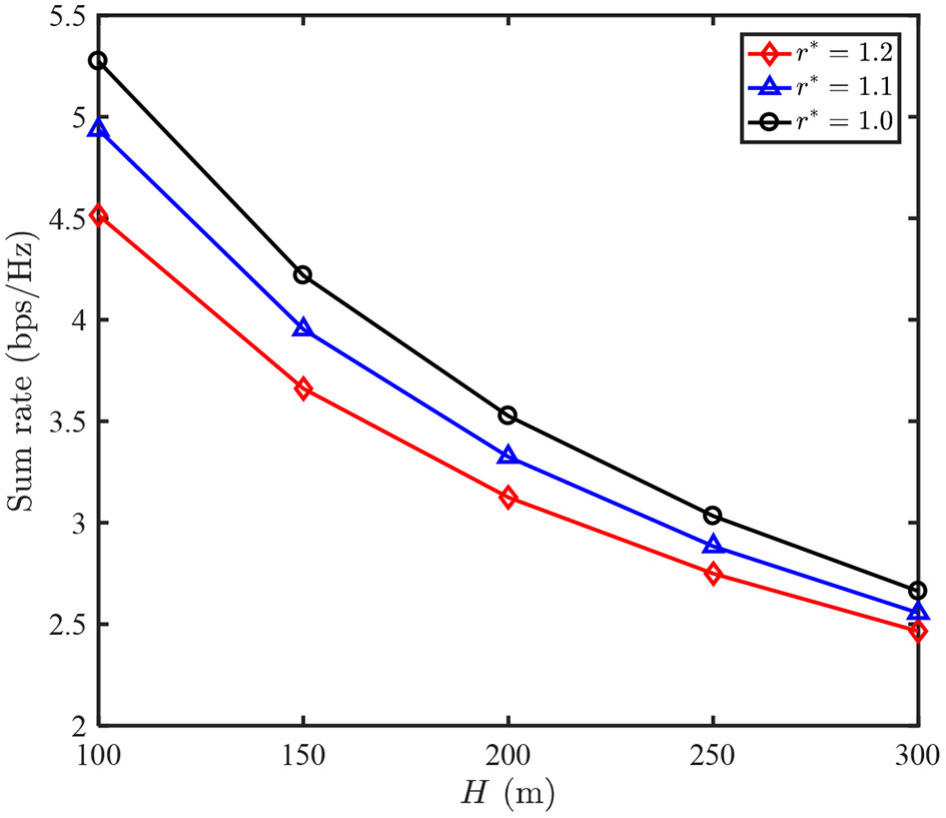
Sum rate of N-LPJO scheme vs. UAV altitude H.

Figure 6 shows the sum rate of our proposed N-LPJO scheme vs. UAV's altitude *H*. It is observed that all the sum rates decrease as *H* increases for each QoS requirement. This is due to the fact that both the channel gains of the two users decrease as *H* increases, hence user 2 has to increase its transmit power for satisfying the QoS requirement, leading to the received power from user 1 decreases. Additionally, we observe that all the performance gains attained by the different r^*^ decrease as *H* increases. This is because that the effect of *H* on the two users' channel gains will be rather small as *H* becomes sufficiently large.

## Conclusions

In this paper, we have investigated an UAV-enabled uplink NOMA system under a total power constraint. To maximize the sum rate of two users, we have demonstrated that the UAV should be deployed at a certain point over the line segment linked by the two users. Then, we have translated the corresponding optimization into a univariate quadratic optimization problem, which can be efficiently solved by our developed scheme. Simulation results showed that our proposed scheme significantly outperforms both the two benchmarks in terms of the sum rate. It should be pointed out that the proposed power allocation policy can be extended to general multi-user systems in a similar way, but extending the proposed UAV deployment scheme to general multi-user systems is not trivial. Our future works will commit to solving the sum rate maximization problem in general multi-user networks.

## Data Availability Statement

The raw data supporting the conclusions of this article will be made available by the authors, without undue reservation.

## Author Contributions

The author confirms being the sole contributor of this work and has approved it for publication.

## Conflict of Interest

The author declares that the research was conducted in the absence of any commercial or financial relationships that could be construed as a potential conflict of interest.

## Footnotes

^1^3GPP. TR 36.777: Enhanced LTE Support for Aerial Vehicles. Available online at: https://www.3gpp.org/ftp/Specs/archive/36_series/36.777 2017.

^2^In problem (P1), we assume that *x* ≥ 0. However, there is also a symmetric solution to problem (P1) for *x* ≤ 0, where the optimal UAV deployment is symmetric with that for *x* ≥ 0 with respect to y-axis, and the optimal transmit power policy reverses.

## References

[B1] Al-Imari M. Xiao P. Imran M. A. Tafazolli R. (2014). “Uplink non-orthogonal multiple access for 5G wireless networks,” in 2014 11th International Symposium on Wireless Communications Systems (Barcelona), 781–785. 10.1109/ISWCS.2014.6933459

[B2] Amoiralis E. I. Tsili M. A. Spathopoulos V. Hatziefremidis A. (2014). Energy efficiency optimization in UAVs: a review. Mater. Sci. Forum 792, 281–286. 10.4028/www.scientific.net/MSF.792.281

[B3] Chen Z. Ding Z. Dai X. Zhang R. (2017). An optimization perspective of the superiority of NOMA compared to conventional OMA. IEEE Trans. Signal Process. 65, 5191–5202. 10.1109/TSP.2017.2725223

[B4] Cui F. Cai Y. Qin Z. Zhao M. Li G. Y. (2018). “Joint trajectory design and power allocation for UAV-enabled non-orthogonal multiple access systems,” in 2018 IEEE Global Communications Conference (GLOBECOM) (Abu Dhabi), 1–6. 10.1109/GLOCOM.2018.8647352

[B5] Dai L. Wang B. Yuan Y. Han S. I. C. Wang Z. (2015). Non-orthogonal multiple access for 5G: solutions, challenges, opportunities, and future research trends. IEEE Commun. Mag. 53, 74–81. 10.1109/MCOM.2015.7263349

[B6] de Sena A. S. Carrillo D. Fang F. Nardelli P. H. J. Costa D. B. Dias U. S. (2020). What role do intelligent reflecting surfaces play in multi-antenna non-orthogonal multiple access? IEEE Wireless Commun. 27, 24–31. 10.1109/MWC.001.2000061

[B7] Ding Z. Lei X. Karagiannidis G. K. Schober R. Bhargava V. A. (2017). Survey on non-orthogonal multiple access for 5G networks: research challenges and future trends. IEEE J. Sel. Areas Commun. 35, 2181–2195. 10.1109/JSAC.2017.2725519

[B8] Ding Z. Yang Z. Fan P. Poor H. V. (2014). On the performance of non-orthogonal multiple access in 5G systems with randomly deployed users. IEEE Signal Process. Lett. 21, 1501–1505. 10.1109/LSP.2014.2343971

[B9] Do D. Nguyen T. Le C. Voznak M. Kaleem Z. Rabie K. M. (2020). UAV relaying enabled NOMA network with hybrid duplexing and multiple antennas. IEEE Access 8, 186993–187007. 10.1109/ACCESS.2020.3030221

[B10] Du J. Wang Z. Fan Z. Wan X. (2020). “Sum rate maximization for UAV-enabled wireless powered NOMA systems,” in 2020 IEEE/CIC International Conference on Communications in China (ICCC) (Chongqing), 753–757. 10.1109/ICCC49849.2020.9238850

[B11] Duan R. Wang J. Jiang C. Yao H. Ren Y. Qian Y. (2019). Resource allocation for multi-UAV aided IoT NOMA uplink transmission systems. IEEE Internet Things J. 6, 7025–7037. 10.1109/JIOT.2019.2913473

[B12] Jiao R. Wang Z. Chu R. Dong M. Rong Y. Chou W. (2020). An intuitive end-to-end human-UAV interaction system for field exploration. Front. Neurorobot. 13:117. 10.3389/fnbot.2019.00117 32116632 PMC7033451

[B13] Liu X. Wang J. Zhao N. Chen Y. Zhang S. Ding Z. (2019). Placement and power allocation for NOMA-UAV networks. IEEE Wireless Commun. Lett. 8, 965–968. 10.1109/LWC.2019.2904034

[B14] Nasir A. A. Tuan H. D. Duong T. Q. Poor H. V. (2019). UAV-enabled communication using NOMA. IEEE Trans. Commun. 67, 5126–5138. 10.1109/TCOMM.2019.2906622

[B15] Saito Y. Kishiyama Y. Benjebbour A. Nakamura T. Li A. Higuchi K. (2013). “Non-orthogonal multiple access (NOMA) for cellular future radio access,” in 2013 IEEE 77th Vehicular Technology Conference (VTC Spring) (Dresden), 1–5. 10.1109/VTCSpring.2013.6692652

[B16] Sharma P. K. Kim D. I. (2017). “UAV-enabled downlink wireless system with non-orthogonal multiple access,” in IEEE Globecom Workshops (GC Wkshps) (Singapore), 1–6. 10.1109/GLOCOMW.2017.8269066

[B17] Sohail M. F. Leow C. Y. Won S. (2018). Non-orthogonal multiple access for unmanned aerial vehicle assisted communication. IEEE Access 6, 22716–22727. 10.1109/ACCESS.2018.2826650

[B18] Timotheou S. Krikidis I. (2015). Fairness for non-orthogonal multiple access in 5G systems. IEEE Signal Process. Lett. 22, 1647–1651. 10.1109/LSP.2015.2417119

[B19] Vaezi M. Amarasuriya G. Liu Y. Arafa A. Fang F. Ding Z. (2019). Interplay between NOMA and other emerging technologies: a survey. IEEE Trans. Cogn. Commun. Netw. 5, 900–919. 10.1109/TCCN.2019.2933835

[B20] Wang Y. Tian Z. Cheng X. (2020). Enabling technologies for spectrum and energy efficient NOMA-MmWave-MaMIMO systems. IEEE Wireless Commun. 7, 53–59. 10.1109/MWC.001.2000055

[B21] Wu Q. Liu L. Zhang R. (2019). Fundamental trade-offs in communication and trajectory design for UAV-enabled wireless network. IEEE Wireless Commun. 26, 36–44. 10.1109/MWC.2018.1800221

[B22] Wu Q. Xu J. Zhang R. (2018). Capacity characterization of UAV-enabled two-user broadcast channel. IEEE J. Sel. Areas Commun. 36, 1955–1971. 10.1109/JSAC.2018.2864421

[B23] Wu Q. Zeng Y. Zhang R. (2017). “Joint trajectory and communication design for UAV-enabled multiple access,” in LOBECOM 2017 - 2017 IEEE Global Communications Conference (Singapore), 1–6. 10.1109/GLOCOM.2017.8254949

[B24] Wu Q. Zhang R. (2017). “Delay-constrained throughput maximization in UAV-enabled OFDM systems,” in 2017 23rd Asia-Pacific Conference on Communications (APCC) (Perth, WA), 1–6. 10.23919/APCC.2017.8304088

[B25] Wu Q. Zhang R. (2018). Common throughput maximization in UAV-enabled OFDMA systems with delay consideration. IEEE Trans. Commun. 66, 6614–6627. 10.1109/TCOMM.2018.2865922

[B26] Zeng Y. Wu Q. Zhang R. (2019). Accessing from the sky: a tutorial on UAV communications for 5G and beyond. Proc. IEEE 107, 2327–2375. 10.1109/JPROC.2019.2952892

[B27] Zeng Y. Zhang R. (2017). Energy-efficient UAV communication with trajectory optimization. IEEE Trans. Wireless Commun. 16, 3747–3760. 10.1109/TWC.2017.268832831627444

[B28] Zeng Y. Zhang R. Lim T. J. (2016). Wireless communications with unmanned aerial vehicles: opportunities and challenges. IEEE Commun. Mag. 54, 36–42. 10.1109/MCOM.2016.7470933

[B29] Zhan C. Zeng Y. Zhang R. (2018). Energy-efficient data collection in UAV enabled wireless sensor network. IEEE Wireless Commun. Lett. 7, 328–331. 10.1109/LWC.2017.2776922

[B30] Zhang N. Wang J. Kang G. Liu Y. (2016). Uplink non-orthogonal multiple access in 5G systems. IEEE Commun. Lett. 20, 458–461. 10.1109/LCOMM.2016.2521374

[B31] Zhao F. Zeng Y. Xi B. (2018). A brain-inspired decision-making spiking neural network and its application in unmanned aerial vehicle. Front. Neurorobot. 12:56. 10.3389/fnbot.2018.00056 30258359 PMC6143798

[B32] Zhao N. Pang X. Li Z. Chen Y. Li F. Ding Z. (2019). Joint trajectory and precoding optimization for UAV-assisted NOMA networks. IEEE Trans. Commun. 67, 3723–3735. 10.1109/TCOMM.2019.2895831

